# Spatiotemporal Heterogeneity of Urban Land Expansion and Urban Population Growth under New Urbanization: A Case Study of Chongqing

**DOI:** 10.3390/ijerph19137792

**Published:** 2022-06-25

**Authors:** Yudan Zhang, Yuanqing Li, Yanan Chen, Shirao Liu, Qingyuan Yang

**Affiliations:** 1 Chongqing Jinfo Mountain Karst Ecosystem National Observation and Research Station, School of Geographical Sciences, Southwest University, Chongqing 400715, China; dandan334296@email.swu.edu.cn (Y.Z.); chenyanan@email.swu.edu.cn (Y.C.); msnlsr@email.swu.edu.cn (S.L.); yizyang@swu.edu.cn (Q.Y.); 2 Chongqing Engineering Research Center for Remote Sensing Big Data Application, Chongqing 400715, China; 3 Key Laboratory of Monitoring, Evaluation and Early Warning of Territorial Spatial Planning Implementation, Ministry of Natural Resources, Chongqing 401120, China

**Keywords:** urban land expansion, urban population growth, spatiotemporal heterogeneity, Chongqing

## Abstract

Land urbanization (LU) and population urbanization (PU) maintain the nature of spatiotemporal heterogeneity in China. As a municipality directly administered by the central government in the mode of “large cities and large rural areas”, Chongqing’s urbanization process is the epitome of China’s urbanization process. This paper examines the spatiotemporal variability of LU and PU in Chongqing on the basis of nighttime light data, the elasticity coefficient of the coupling relationship, and GWR. The results show that (1) the urban land and urban population in Chongqing grew notably from 2008 to 2018, with average annual growth rates of 9.4% and 2.3%, respectively. (2) The coupling coordination coefficient of LU and PU in Chongqing was 0.24, and the total number of districts and counties with uncoordinated development increased, but the overall uncoordinated situation gradually improved over the period. (3) The influence of PU on LU in each district and county increased year by year, and it showed a decreasing trend from southwest to northeast in Chongqing, which indicates that LU was increasingly adapted to the construction needs of PU. The gap between LU and PU widened due to the household registration system, land fiscal policies and other policies. After the reform of the household registration system and the adjustment of new pilot policies targeting the construction of new-type urbanization, the coupling relationship between LU and PU was gradually improving to the coordinated mode. The findings indicate that Chinese urban areas should adhere to the principle of new-type urbanization construction and carry out scientific land planning strategies, strictly controlling land expansion to promote the reasonable development of population growth.

## 1. Introduction

Urbanization represents a fundamental change in a region’s economic and social structure and the style of production and life, which involves land urbanization (LU) and population urbanization (PU) [1,2]. According to the United Nations’ population survey report, urban population growth is the main reason for urban land expansion [3]. Currently, more than half of the global population lives in urbanized areas [4]. The global population is predicted to arrive at 9.7 billion in 2050, and more people will move into cities. After the reform and opening up, China’s urbanization process has gradually accelerated, and many urban problems have emerged, such as land expansion, resource waste, excessive expansion of large cities, and slow development of small cities [5,6]. LU is an active process of changing the ratio of a built-up area and the total urban area [7]. Rapid land expansion has brought plenty of fiscal revenues for China, providing much funding to support economic activities and infrastructure construction [8]. However, urban expansion mainly on the basis of land occupation also causes the extensive use of construction lands with low land use efficiency, which also intensifies the contradiction of farmland protection and urban expansion [9,10,11]. The PU is a process in which the proportion of the urban population in the total population increases due to the continuous flow of the nonurban population to urban areas [12]. It is predicted that China’s PU rate will reach 70% in 2030 [13]. As the direct driving force of LU [14], the urban population increases the demand for urban construction and further promotes urban expansion. As the key to expanding the domestic market, the urban population is crucial in changing the economic development mode. In 2014, China launched a pilot program of new-type urbanization, aiming to achieve the goal of economical and intensive urbanization, and coordinating the relationship between PU and LU [15].

Once the imbalance of LU and PU arises, it will harm the sustainable development of a city [16]. Under China’s rapid urbanization, the relationship between the rapid expansion of urban land and the rapid population growth has attracted extensive attention from scholars in China and abroad [17]. Existing studies show that the current LU in China is more than the PU [18]. This shows that the gap between urban and rural areas is further expanding, and the urban areas are overdeveloped, reducing the arable land area and resulting in a decline in land fertility in rural areas. Because LU and PU play complementary roles [19], numerous recent studies on urban land expansion and urban population growth have focused on the coupling coordination of the two [20,21]. It is feasible to take the coupling elasticity coefficient of PU and LU to describe the relationship between the two. Shen et al. [22] analysed the spatiotemporal evolution features of urban land and population in Africa from 2000 to 2019 by using the elasticity index. They concluded that the development trend of urban land and population across Africa in the past 20 years was not coordinated. Wu et al. [23] conducted a coordinated analysis of the data on the LU and PU of 636 administrative cities in China from 2006 to 2014 using the elasticity coefficient. They concluded that the misalignment phenomenon was relatively common. However, this kind of research does not extract different coordination types at different spatial and temporal levels. Most studies take the country or provincial capital as a whole [24]. The scope of such studies is broad, and they cannot easily reflect the features of local areas. The statistical data of built-up areas cannot meet the needs of researching urban spatial expansion. The continuous development of remote sensing and geographic information technology improved conditions for obtaining information on the urban land scale in the time-space continuum, enabling a more accurate portrayal of urban physical areas [25]. Since the correlation between population and LU is a spatial relation, if the two were assumed to be fixed [26], we would use the traditional linear regression method for analysis, but this would violate the heterogeneity of spatial relations in the real world, the principle of nonstationarity, and the first law of geography [27]. Consequently, we attempted to put forward a technique to accurately identify LU and PU and objectively evaluate their spatiotemporal heterogeneity.

The objectives of the study were as follows: (1) to explore the changes in the urban expansion and population growth of districts and counties in Chongqing from 2008 to 2018; (2) to analyse the spatiotemporal heterogeneity of the PU and LU in Chongqing; and (3) to prove that the impact of PU on LU in Chongqing has spatiotemporal heterogeneity. The spatial organization form of Chongqing maintained the pattern of “large cities and large rural areas” [28] and presented an urban–rural dual structure [29]. The development conditions of Chongqing’s layout of “one urban area and two town groups” are highly similar to the development patterns of the eastern and western regions of China. This study aims to study urbanization features in this region and provide a reference for rational land use, population policy setting, and new urbanization development.

## 2. Materials and Methods

### 2.1. Study Areas

**Figure 1:**
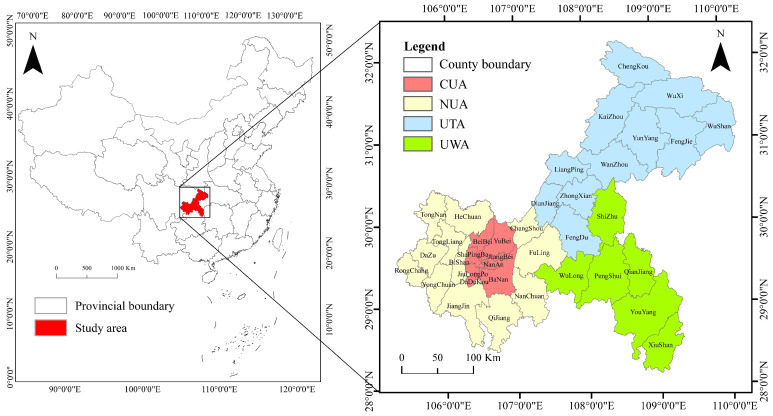
Map of the research area.

As the case area for the study, this paper selects Chongqing, which has a total area of 8.24 × 10^4^ km^2^ and covers 38 districts and counties under its jurisdiction, including 26 districts, 8 counties and 4 autonomous counties. In 2021, Chongqing had a permanent population of 3.21 × 10^7^ and a PU rate of 70.32%. The Chengdu-Chongqing Economic Circle is also the fourth pole of growth in China, such that it is the region with the densest population, the most robust industrial foundation, and the highest degree of opening up in western China. Chongqing established the development pattern of “one urban area and two town groups” (OATG) in 2014 (Figure 1). That is, the city proper (CP) of Chongqing drives the development of the urban clusters of the Three Gorges Reservoir area (UTA) and the urban clusters of the Wuling mountainous area (UWA). In 2020, Chongqing expanded its CP from 9 to 21 districts, including the central urban areas (CUA) of Yuzhong, Dadukou, Jiangbei, Shapingba, Jiulongpo, Nan’an, Beibei, Yubei, and Ba’nan, as well as the 12 districts of new downtown urban areas (NUA): Changshou, Jiangjin, Bishan, Nanchuan, Changshou, Jiangjin, Bishan, Nanchuan, Dazu, Tongliang, Tongnan, and Rongchang. Due to different natural and economic conditions, the CP, UTA, and UWA in Chongqing have different functional orientations. This paper takes the prefecture-level administrative division in 2018 as the criteria and the 38 districts and counties in Chongqing as the research objects.

### 2.2. Data Sources

Defense Meteorological Satellite Program/Operational Linescan System (DMSP/OLS) nighttime light data, National Polar—orbiting Partnership/Visible Infrared Imaging Radiometer Suite (NPP/VIIRS) nighttime light data, the impervious surface area (ISA) data, population density data, statistical data, and administrative boundaries were used in this study (Table 1).

The DMSP/OLS (2008–2013) and NPP/VIIRS (2013–2018) data were collected and processed by (NOAA/NGDC). The DMSP/OLS data values ranged from 0 to 63 with a spatial resolution of 30 arc-seconds. The NPP/VIIRS data involved a boosted spatial resolution of 15 arc-seconds. We calibrated the DMSP/OLS (2008–2013) by the “pseudo-invariant pixels” method, and predicted and patched the missing pixels in NPP/VIIRS (2013–2018) using the exponential smoothing model. We converted NPP/VIIRS into DMSP/OLS-like data (2013–2018) through a sigmoid model, and calculated it with the calibrated DMSP/OLS (2008–2013) to generate this nighttime light (NTL) dataset (2008–2018). It was proven that this NTL dataset can solve the problem of the two sets of night light data not being able to be used together, and can be utilized for long-time series research of urban problems with high accuracy [30].

The population data came from the statistical data of Chongqing over the years. Most studies on PU in China are based on data on the PU rate of permanent residents [31]. Therefore, this paper refers to the permanent urban population with sound continuity as an indicator to measure the urban population scale, as many urban studies have done. The permanent resident population data in 38 districts and counties from 2008 to 2018 were obtained on the basis of the administrative divisions in 2018 in consideration of the change from county to district, which ensured the continuity of the permanent urban population data.

The population density data were derived from the accurate population data mapped by the WorldPop project at the University of Southampton in the UK by large-scale data processing in Microsoft Azure, which was matched with the population estimate from the United Nations to obtain the population dataset of Chongqing from 2008 to 2018.

The ISA data (2008–2018) were extracted from China’s Tsinghua database with a spatial resolution of 30 m, and the ISA was closely related to human activities. In this paper, the data were preprocessed by cutting.

The administrative boundary vector data of districts and counties of Chongqing in 2018 were obtained from the China National Geographic Information Center.

All spatial data in this study were resampled to a resolution of 250 m and were converted in Albers equal area projection with reference to the WGS-84 coordinate system.

### 2.3. Methods

**Figure 2:**
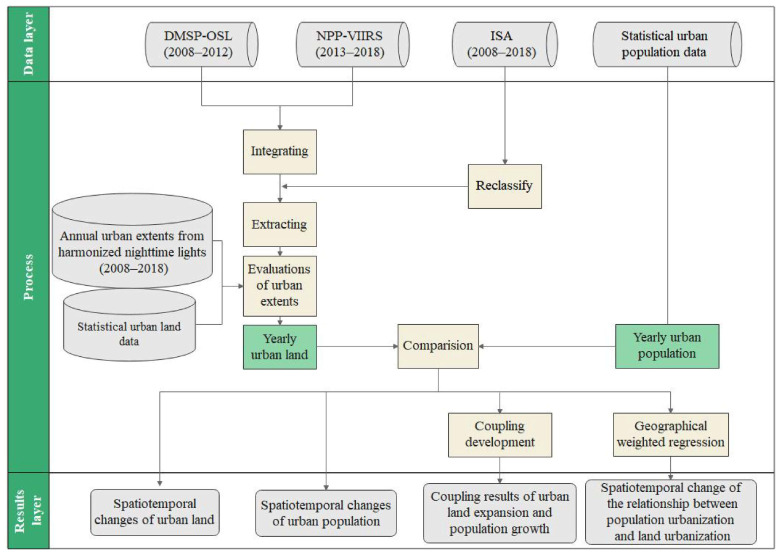
Flowchart and technical framework of the research.

The designed technical framework involved 4 steps (Figure 2): (1) utilize NTL data and ISA data to extract the expansion conditions of urban land in Chongqing from 2008 to 2018; (2) use urban population statistics to determine the urban population change laws in space and time; (3) analyse the temporal and spatial differentiation of PU and LU by the coupling elasticity coefficient; (4) prove and analyse that the impact of PU on LU in Chongqing has spatiotemporal heterogeneity.

#### 2.3.1. Extraction of Built-Up Areas

In the study of urbanization, it is important to acquire accurate built-up area information. DMSP/OLS is an important indicator reflecting the level of urbanization and is widely used in urban mapping within local regions [32,33] since Croft [34] first proposed the feasibility of using DMSP/OLS to extract urban built-up areas. However, under the influence of saturation diffusion, threshold selection, and other problems, urban areas cannot be identified only by NTL data [35]. Impermeable substances, such as lanes, sidewalks, parking lots, and roofs [36], are the main components of urban built-up areas, which are identified and used in built-up area extraction. In this paper, we comprehensively applied the preprocessed night light time series data and ISA data to obtain the built-up areas of 38 districts and counties in Chongqing from 2008 to 2018 by using the ISA data from 2008 to 2018 as the mask of urban areas. After extracting the built-up areas of each district and county, we used statistical yearbook data and the city scope generated by Zhao et al. [37] to prove that the accuracy of the built-up areas extracted in this paper meets the requirements qualitatively and quantitatively.

#### 2.3.2. The Coupling Development Analysis of LU and PU

To judge whether there are differences between the development and speed in LU and PU and to estimate whether the mutual relationship is coordinated or not, the classification method of the elasticity coefficient of the coupling relationship between LU and PU was adopted, based on the studies on the changes in agricultural population and settlement types performed [38] by Wu et al. [23].

The elasticity coefficient is calculated as follows:

(1) $$\mathrm{EC}=\frac{\mathrm{PR}}{\mathrm{LR}},$$

where *EC* represents the coupling elasticity coefficient of PU and LU. *PR* is the average annual rate of PU, and *LR* is the average annual change rate of LU.

**Figure 3:**
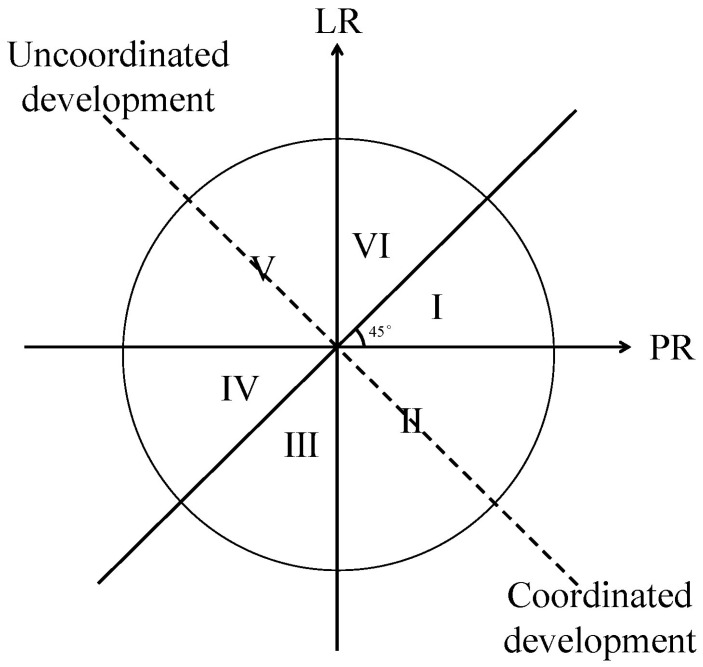
The classification of different coupling relationship types.

As shown in Figure 3, the coupling relationship between population and land can be divided into six types I–VI based on the scale comparison of *PR* and *LR*, including the coordinated coupling relationships of Type “I–VI ”, which result along with the development under three conditions: simultaneous increases in population and land (Type I), increase in population and decrease in land (Type II), and simultaneous decrease in both population and land (Type III), respectively. In contrast, under the same three development modes, there can be uncoordinated development conditions (Type IV–VI). When the growth rate of PU is higher than that of LU, or the decline rate of PU is lower than that of LU under the coordinated coupling model, the people–land relationship is more harmonious. For cities with uncoordinated features, the growth rates of PU are lower than those of LU, while the decline rates of PU are higher than those of LU, which presents the imbalance of growth between them. In this paper, the proportion of permanent urban population in the total resident population was used to depict the PU, and the proportion of built-up area in the areas of administrative divisions was used to depict the LU.

#### 2.3.3. Spatial Autocorrelation Analysis

According to the first law of geography and spatial dependence [39], the overall Moran’s I indexes were selected to test the spatial correlation of PU in 38 districts and counties [40]. The calculation formula is as follows [41]:

(2) $$I=\sum_{i=1}^{n}\sum_{j=1}^{n}(x_{i}-\bar{x})/s^{2}\sum_{i=1}^{n}\sum_{j=1}^{n}W_{\mathrm{ij}},$$

where $s^{2}=\sum_{i=1}^{n}\frac{{\left(x_{i}-\bar{x}\right)}^{2}}{n}$, $x_{i}$ is the observed value of prefecture-level units, and $W_{\mathrm{ij}}$ is the spatial weight matrix. Where Moran’s *I* > 0 indicates that the data have a positive spatial correlation. Moran’s *I* < 0 indicates that the data have a negative spatial correlation. 

This paper uses the exploratory data analysis method to analyse the local Moran index of PU from 2008 to 2018 [42]. The *Z-score* value significance test for Moran’s *I* value is carried out. When the Z-score value is negative and obvious, it indicates that a “high-low (H-L)” or “low-high (L-H)” agglomeration occurs. When the Z-score value is positive and obvious, it indicates a “high-high (H-H)” agglomeration or “low-low (L-L)” agglomeration in the region.

#### 2.3.4. GWR Analysis 

Brunsdon et al. [43] proposed the GWR model in 1996 after the local weighted regression analysis model was developed, which focused on spatial heterogeneity. This model inserts the geographical location of sample points into regression parameters and supplements the traditional linear regression model [44]. This method can calculate the local estimate value of the function given by each geographical location, so it can intuitively present the spatial changes in the estimated values of spatial heterogeneity factors [45] at the scale of each county in Chongqing. Therefore, this paper adopts this model to reflect the local spatial changes from the impact of PU on LU and adheres the minimum Aic_c_ value principle to select the optimal bandwidth [46].

The model structure is as follows:

(3) $$y_{i}{=\beta }_{0}\left(u_{i}{,v}_{i}\right)+\sum_{k}\beta_{k}\left(u_{i}{,v}_{i}\right)x_{\mathrm{ik}}{+\varepsilon }_{1},$$

where $\left(u_{i},v_{i}\right)$ is the geographic centre coordinate of the first sample spatial unit and $\beta_{k}\left(u_{i},v_{i}\right)\;$is the value of the continuous function of $\beta_{k}\left(u_{i},v_{i}\right)$ in the sample spatial unit i, which is related to the geographical location.

## 3. Results

### 3.1. Built-Up Area Verification

**Figure 4:**
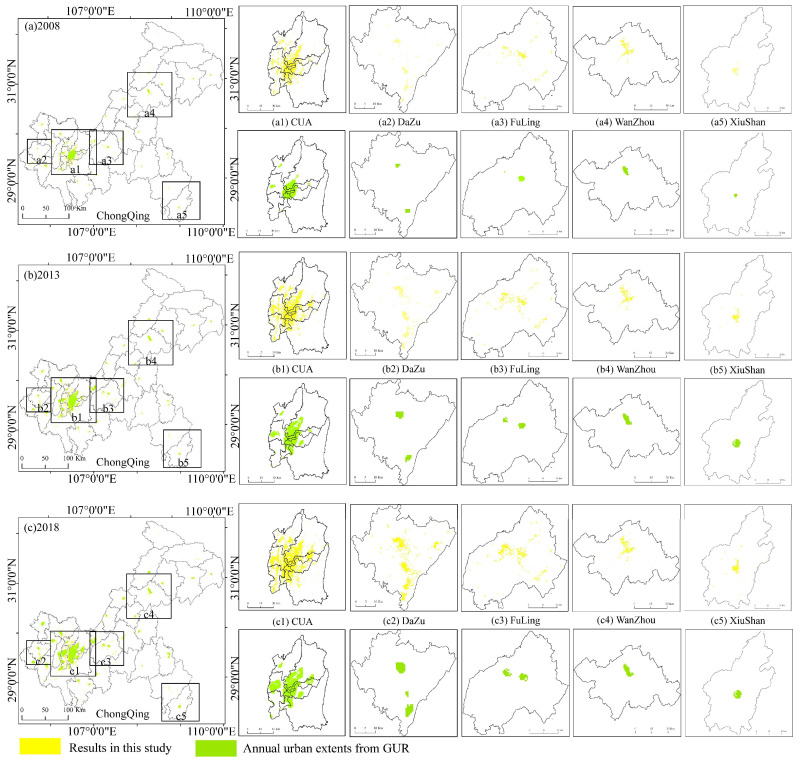
Comparison of the spatial range between the results of the built-up areas extracted in this paper and the dataset of the GUR. The spatial range comparison of the two datasets in 2008 (**a**), 2003 (**b**), and 2018 (**c**) in Chongqing. The comparison of the two datasets in CUA (**a1**,**b1**,**c1**), Dazu (**a2**,**b2**,**c2**), Fuling (**a3**,**b3**,**c3**), Wanzhou (**a4**,**b4**,**c4**), and Xiushan (**a5**,**b5**,**c5**) in 2008, 2013, and 2018.

**Figure 5:**
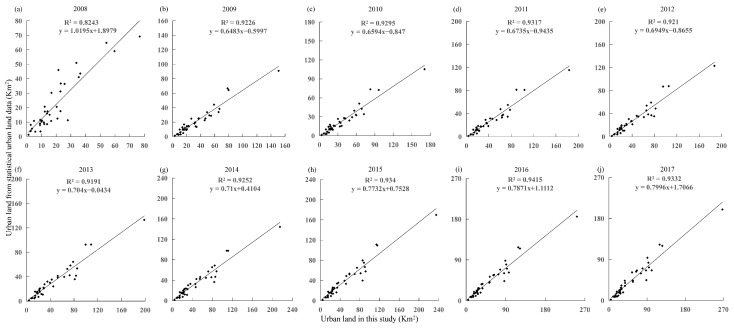
The results of the built-up area extracted in this paper are compared with statistical data. (**a**–**j**) The linear regression results of the extraction of the built-up area in this paper and the statistical data of the urban built-up area during 2008–2017.

To test the feasibility of extracting the built-up area in this paper, we compared it with the data of the global urbanization region (GUR) from 1992 to 2020 generated by Zhao et al. [37] for qualitative verification. These data were proven to offer a reliable representation of the city scope. We used artificial visual judgement to determine the spatial location error between two built-up area datasets. We selected five representative urban built-up areas (CUA, Dazu, Fuling, Wanzhou, and Xiushan) as comparison areas from CP, UTA, and UWA. The data from 2008, 2013, and 2018 were used for comparison. As shown in Figure 4, the time series of urban areas extracted from five regions of different types in Chongqing and the images of built-up areas extracted from GUR data overlap in space with good consistency. The extracted built-up area boundary is fragmented, which meets the needs of researching the expansion of land use scale in county-level cities. This paper used the sum of urban areas and built-up town areas from statistical data for quantitative comparative analysis. Due to the lack of statistical data sources in 2018, the data from 2008 to 2017 were selected for linear regression comparison with the extracted built-up area. The results show (Figure 5) that R^2^ over the decade was between 0.82 and 0.92, with an apparent linear trend, indicating that the time dynamics of the built-up area extracted from NTL data tended to be consistent with that of the built-up area in the statistical yearbook.

### 3.2. Time Series Change and Spatial Distribution of Urban Land

The built-up area of Chongqing increased by 1037 km^2^ from 2008 to 2018, with an increase of 2.68 × 10^2^ km^2^ from 2008 to 2011, 3.05 × 10^2^ km^2^ from 2011 to 2014, and 2.63 × 10^2^ km^2^ from 2014 to 2018. In 2018, the total built-up area of Chongqing reached 1.75 × 10^3^ km^2^, indicating the overall rapid growth of Chongqing’s built-up area, with an average annual growth rate of 9.46.

**Figure 6:**
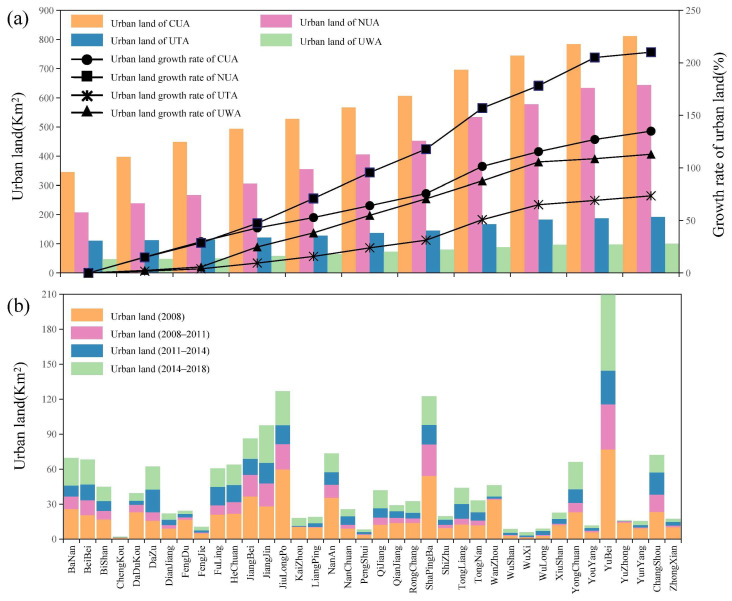
Spatial–temporal changes in land use in Chongqing during 2008–2018. (**a**) Total amount and growth rate of urban land in Chongqing’s OATG area (CUA, NUA, UTA, and UWA) during 2008–2018. Note: The growth rate is based on 2008. (**b**) The total amount of urban land in 38 districts and counties of Chongqing in 2008 and the increase in urban land during 2008–2011, 2011–2014, and 2014–2018.

Chongqing’s urban land use growth had different spatial characteristics in the long-term time series. As shown in Figure 6 and Table 2, the development of the built-up area of Chongqing was mainly concentrated in the CP. The total amount and increase rate of urban land in the UTA and UWA were lower than those in the CUA and NUA. Based on 2008, this paper estimates the growth rate of urban land in each region to intuitively reflect the difference in the rates across regions. The growth rate of urban land in the CUA was lower than that in the NUA. From 2008 to 2011, the growth rate of urban land in the NUA was the highest in the city, at 14%. The rapid growth made the total built-up area of the NUA and CUA maintain a trend of general development. Although the total amount of urban area in the UWA was lower than that in the UTA, it showed a high growth rate, with an annual growth rate of 8%. 

**Figure 7:**
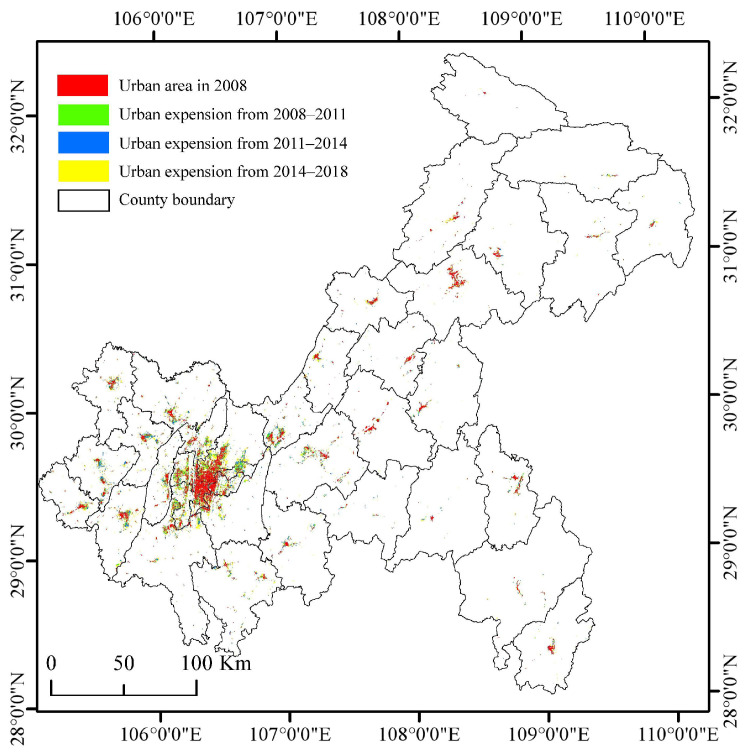
Map of the urban land expansion of districts and counties in Chongqing during 2008–2018, including urban land area in 2008 and urban land growth area during 2008–2011, 2011–2014, and 2014–2018.

As shown in Figure 7 and Table 2, there were differences in the expansion of built-up areas within each region. Among the CUA, Yubei had the highest built-up area, reaching 2.10 × 10^2^ km^2^ in 2018 and accounting for 25.8% of the total built-up area of the CUA, far higher than other districts of the CUA. The three districts with the fastest growth rates were Beibei, Yubei, and Banan, with average annual growth rates ranging from 11 to 13% during 2008–2018. In contrast, the growth rate of urban land in Dadukou was relatively low, with an average annual growth rate of 6%. Due to the high degree of urbanization, the built-up area of Yuzhong had increased by less than 2 km^2^, which reflects that its LU process was very different from the rapid LU of other areas. In the NUA, due to the different bases for urban development, some districts and counties had higher built-up areas in 2008, among which JiangJin and Changshou had 27.93 km^2^ and 23.13 km^2^, while Nanchuan had the smallest built-up area, with only 9.06 km^2^. After ten years of development, the built-up area of Jiangjin had the most significant growth, with a total increase of 69.56 km^2^. The growth of the built-up area in Changshou was greater than 40 km^2^. The built-up area of Dazu had the highest growth rate, at 15%. Rongchang had the lowest annual growth rate, at 9%.

In the UTA, the built-up area of Wanzhou was much higher than that of other districts and counties in the same region. In 2008, the built-up area of Wanzhou was 33.75 km^2^, while the built-up area of most areas in the UTA was very small, with 1.25 km^2^ in Chengkou, 4.93 km^2^ in Fengjie, and 2.75 km^2^ in Wushan. During the ten years, the built-up area of Wanzhou increased by 46.19 km^2^, but its annual growth rate was only 3%, which was the lowest in the UTA. Although the built-up area of Wuxi was still less than 10 km^2^, the annual growth rate of Wuxi was 16%, the highest in the region. The size of the built-up area in the UTA decreased from southwest to northeast. In the UWA, only Qianjiang and Xiushan had built-up areas exceeding 10 km^2^ in 2008. The built-up areas of other districts and counties were lower, with 3.56 km^2^ in Pengshui and 2.5 km^2^ in Wulong. From 2008 to 2018, the built-up areas of Qianjiang and Xiushan reached 28.94 km^2^ and 22.56 km^2^, respectively. Pengshui and Wulong had higher growth rates of built-up areas, with 9% and 14%. In 2018, the built-up areas of Pengshui and Wulong were 8.13 km^2^ and 8.88 km^2^, with the total built-up area still at the lowest level in the city.

### 3.3. Time Series Change and Spatial Distribution of Urban Population

From 2008 to 2018, the urban population of Chongqing increased by 6.85 × 10^6^ people, including 1.85 × 10^6^ people from 2008 to 2011, 2.16 × 10^6^ people from 2011 to 2014 and 2.84 × 10^6^ people from 2014 to 2018. In 2018, the total urban population of the city was 2.10 × 10^7^. The growth rate of the urban population was lower than the growth rate of urban land expansion, with an average annual growth rate of 4.02%.

**Figure 8:**
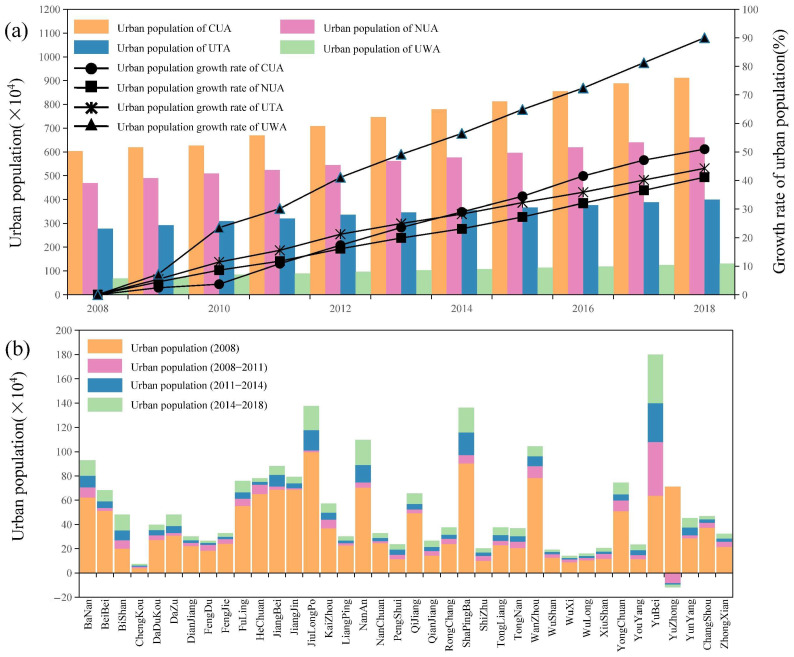
Spatial–temporal changes in the urban population in Chongqing during 2008–2018. (**a**) Total amount and growth rate of the urban population in Chongqing’s OATG area (CUA, NUA, UTA, and UWA) during 2008–2018. Note: The growth rate is based on 2008. (**b**) The total urban population in the 38 districts and counties of Chongqing in 2008 and the urban population growth during 2008–2011, 2011–2014, and 2014–2018.

**Figure 9:**
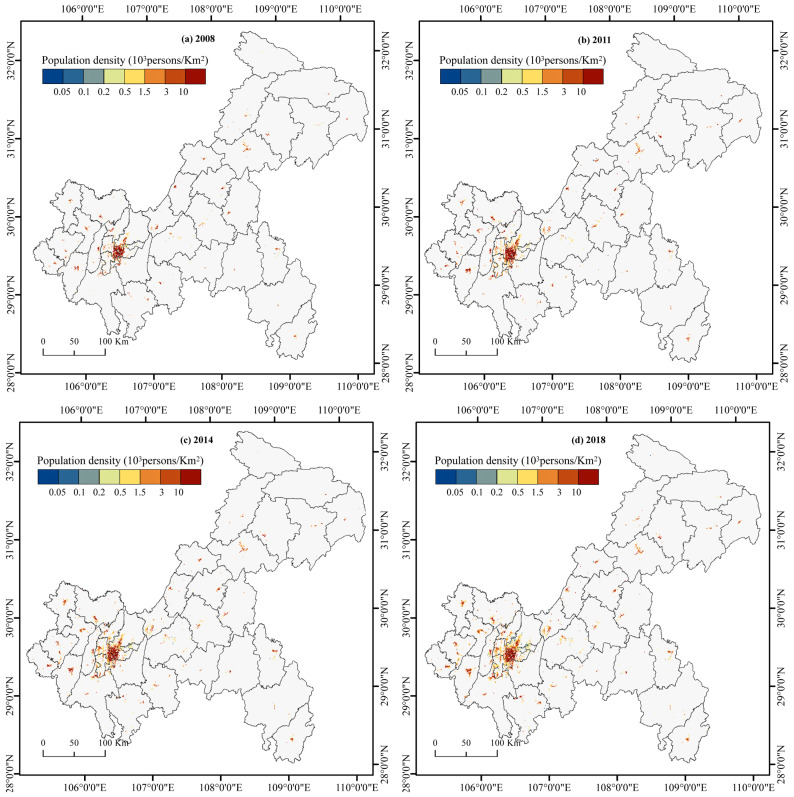
Spatial distribution of the population density of Chongqing in 2008 (**a**), 2011 (**b**), 2014 (**c**), and 2018 (**d**).

As shown in Figure 8 and Table 3, the urban population growth in Chongqing had apparent differences in regional distribution in 2008–2018. The total urban population in CUA increased by 3.08 × 10^6^ people. We also evaluated the growth rate of the urban population in each region based on the level in 2008. From 2008 to 2018, the UWA and CUA had the fastest growth rate in urban population. The gap between the CUA and the NUA and between the UTA and the UWA gradually widened. In 2018, the urban population of the CUA was 9.12 × 10^6^, accounting for 43.34% of the urban population of Chongqing. Although a strong agglomeration effect was reflected, the degree of convergence of the urban population to the CUA was slightly lower than that of urban land expansion. The growth rate of the NUA was lower than that of other regions, with an increase of 1.92 × 10^6^ people over the decade, far higher than the increases in the UTA and UWA. From 2008 to 2018, the urban population in the UTA increased by 1.23 × 10^6^ people, but the growth rate was low compared with that in other districts and counties of the city. The urban population rate in the UWA was higher than that in the other three regions, and the annual average growth rate reached 9%. In terms of total population, the total urban population of the UWA was 1.31 × 10^6^ in 2018, accounting for only 6.21% of the urban population of Chongqing. As shown in Figure 9, due to the large base and a large amount of urban population growth, the population density inside the CP gradually increased. The areas with population density >1500 people/km^2^ expanded to the surrounding areas, with Yuzhong as the centre. The population density decreased in the UTA and UWA, which shows that the spatial structure of the urban population was generally concentrated.

The characteristics of urban population growth are slightly different within the OATG (Table 3). In 2018, the urban population of Yubei was 1.80 × 10^4^ people, accounting for 19.74% of the total urban population of the CUA. The growth rate of the urban population in Yubei was significantly higher than that of the other CUA, with an annual average growth rate of 11%. The annual average growth rate of Nanan, which ranked second in the CUA, was only 5%. In contrast, Yuzhong was the only district with a continuous decline in urban population from 2008 to 2018, with an average annual decline rate of 2%, reflecting the characteristics of urban contraction. In the NUA, Bishan had a significantly higher urban population growth rate than the other areas, with an average annual urban population growth rate of 9%. Tongnan and Dazu maintained growth of approximately 5% a year. Hechuan and Jiangjin had high total urban populations and low growth rates. The growth rate in Changshou was at a low level compared with the other areas. In the UTA, the average annual growth rate of the urban population of each district and county was more than 3%, which is a high growth rate, but the total amount of many districts and counties was low. The average annual growth rate of the urban population of Chengkou was 7%, but it continued to have the lowest urban population in Chongqing. In 2018, the urban population of Wanzhou accounted for 26.15% of the UTA and maintained an annual growth rate of 3%. Although Liangping, Dianjiang, and Wanzhou were close to the NUA, the annual growth rate of the urban population was lower than the average level of the UTA. In the UWA, the average annual growth rate of the urban population of each district and county was higher than 5%, among which the total urban population of Wulong was 1.03 × 10^5^ people in 2008. The average annual growth rate of the urban population was 5%, which was lower than that of other areas. In general, the UWA showed faster growth characteristics than the other regions. However, due to the low total urban population of each district and county, the higher growth rate was not enough to reduce the gap in the total urban population of Chongqing.

### 3.4. The Relationship between Urban Land and Urban Population

#### 3.4.1. Spatiotemporal Characteristics of LU and PU Coupling

From 2008 to 2018, the elasticity coefficient of the coupling relationship between the overall urban population and urban land in Chongqing was 0.24, and the coupling relationship was located in zone VI. 

**Figure 10:**
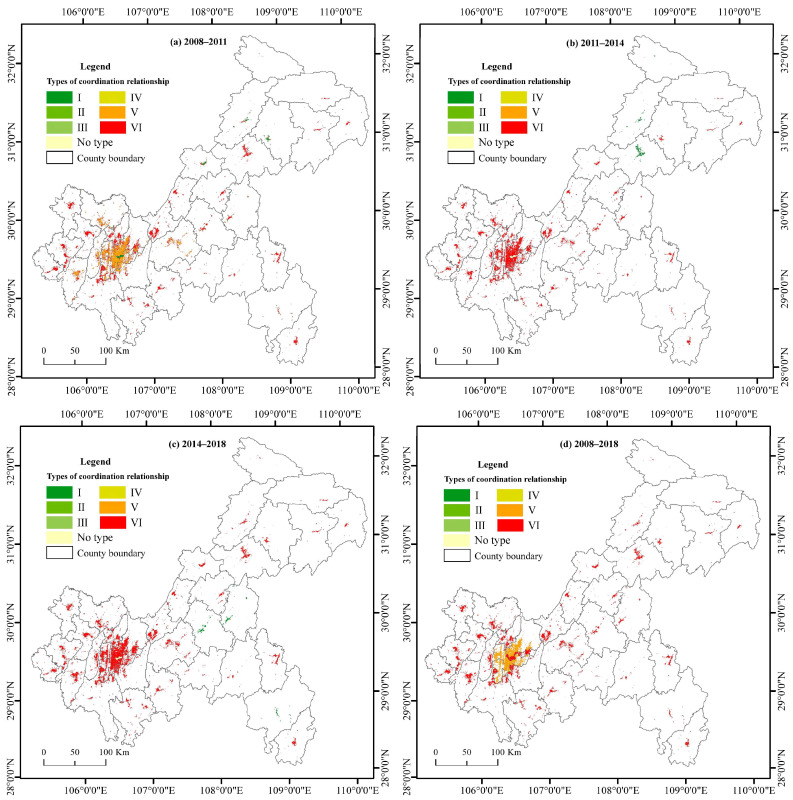
Spatial distribution of coupling linkages in different periods in Chongqing city. The spatial distribution of coupling types of districts and counties in Chongqing during 2008–2011 (**a**), 2011–2014 (**b**), 2014–2018 (**c**), and 2008–2018 (**d**).

As shown in Figure 10, from 2008 to 2011, the primary type of urbanization development in Chongqing was type VI, and the number of districts and counties with type I coordinated urbanization development was much higher than that of 2011–2014 and 2014–2018. The districts and counties with coordinated development were Fengdu, Fengjie, Kaizhou, Liangping, Wanzhou, Wuxi, Yunyang, and Zhongxian in the UTA; Pengshui, Shizhu, and Youyang in the UWA; and Tongnan in the NUA. In this period, type V appeared; that is, the urbanization rate of the population decreased, while the urbanization rate of land increased in the CUA, which were Dadukou, Jiangbei, Jiulongpo, Nanan, and Shapingba. In 2011–2014, only Kaixian and Wanzhou in the UTA remained in the coordination state among the districts and counties with type I coordination in the last period. During this period, Chengkou came to present the type I coordinated mode. The uncoordinated mode VI accounted for the majority of the city areas. From 2014 to 2018, all three areas that were previously type I changed to type VI, and Fengdu, Shizhu, and Youyang became areas of coordinated development, while the rest of the areas still showed uncoordinated characteristics. From the calculation of the coupled elasticity coefficient of LU and PU during the whole period from 2008 to 2018, we find that only the coordination of the simultaneous increase in urban people with urban land occurred due to the continuous improvement of the LU rate in each district and county from 2008 to 2018. The number of cities with coordinated development gradually decreased over time. The type of imbalance in Chongqing was mainly the unbalanced development mode of the simultaneous growth of the urban population and urban land. This mode maintained an absolute position in the process of urbanization for 10 years, and most districts and counties showed this development mode. The most significant gap was in Jiangjin, where the speed of LU was 13.84 times faster than the speed of PU. 

#### 3.4.2. Spatial Correlation Analysis of PU

**Figure 11:**
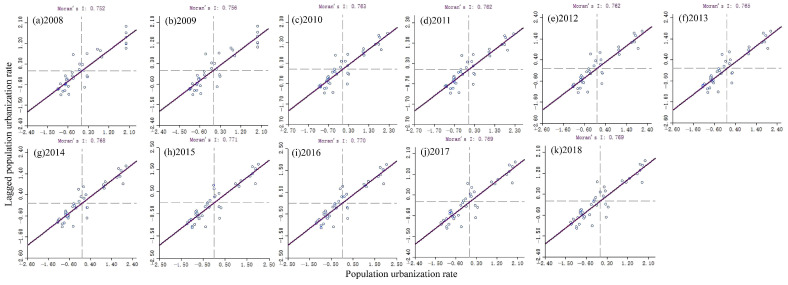
The results of the Spatial Correlation Analysis of PU. (**a**–**k**) Moran Index of PU during 2008–2018.

The spatial distribution of PU is not independent and has spatial correlation, which is the premise of GWR analysis. This study analysed the spatial correlation of PU from 2008 to 2018. Based on this, it was concluded that the Moran index of PU was between [0.75 and 0.77]. The global Moran’s I scores from 2008 to 2018 were all positive, and all *p* values were ≤0.05. This shows that the distribution of PU levels in Chongqing had a significant positive spatial correlation and spatial aggregation. As shown in Figure 11, the global Moran’s I score first increased and then decreased over the studied decade, reaching its peak in 2015. It showed an overall upward trend, indicating that the variable of permanent urban population was increasingly concentrated in areas with advanced urban development foundations and economic development conditions.

**Figure 12:**
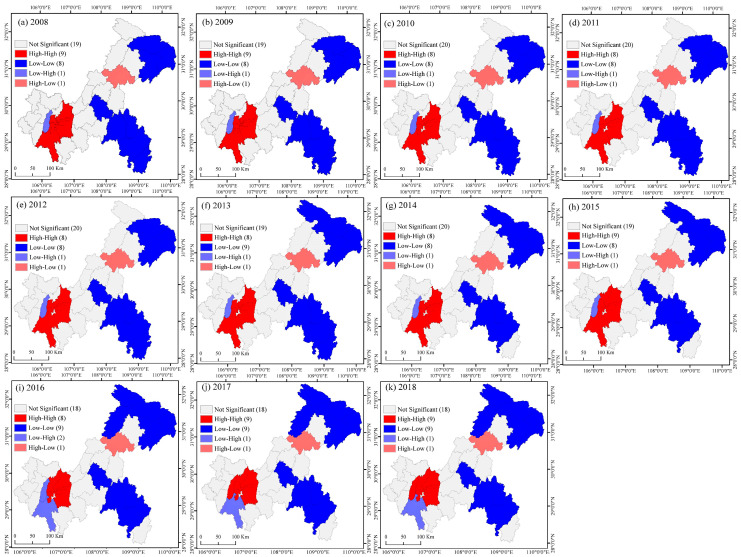
The local spatial autocorrelation of the PU. (**a**–**k**) LISA diagram of PU in Chongqing during 2008–2018.

The local Moran’s I index is adopted to further analyse the local spatial autocorrelation of the PU of each district and county in Chongqing. According to the local Moran’s I index, all districts and counties were divided into four spatial patterns of “H-H”, “L-L”, “H-L”, and “L-H”, and LISA clustering maps of each time section from 2008 to 2018 were obtained (Figure 12). The calculated results all passed the *p* value ≤ 0.05. Four spatial patterns appeared in 2008–2018. Among them, “H-L” appeared in Wanzhou, indicating that Wanzhou’s PU was much higher than that of surrounding districts and counties. The “L-H” cluster indicates that the PU level of the district and county was lower than that of the surrounding districts and counties, and this feature appeared in Bishan and Jiangjin. From 2008 to 2016, the PU level of Bishan was lower than that of surrounding areas of the CUA. After 2016, the PU level of Bishan came to present “H-H” agglomeration, indicating that the PU level of Bishan district gradually converged with the level of the CUA. In contrast, after 9 consecutive years of “H-H” agglomeration, the PU level of Jiangjin gradually became lower than that of the neighbouring districts and counties, and it came to present “L-H” agglomeration. From 2008 to 2018, the “H-H” agglomeration continuously occurred in Yubei, Jiangbei, Shapingba, Jiulongpo, Yuzhong, Nanan, and Banan. After 2015, Beibei showed characteristics of “H-H” agglomeration, and Dadukou showed high agglomeration in 2008–2009, but the PU level did not form an agglomeration effect with the surrounding CUA. The “L-L” agglomeration type was concentrated in the UTA and UWA. In 2008, Wuxi, Wushan, and Fengjie showed “L-L” agglomeration. Chengkou was added in 2013, and Kaizhou was added in 2016. The areas of “L-L” agglomeration gradually expanded. In 2008, Fengdu, Pengshui, Qianjiang, Youyang, and Xiushan were “L-L” agglomeration areas in the UWA. This region’s economy was making it relatively challenging to promote PU. 

#### 3.4.3. The Spatiotemporal Heterogeneity under the Impact of PU on LU

**Figure 13:**
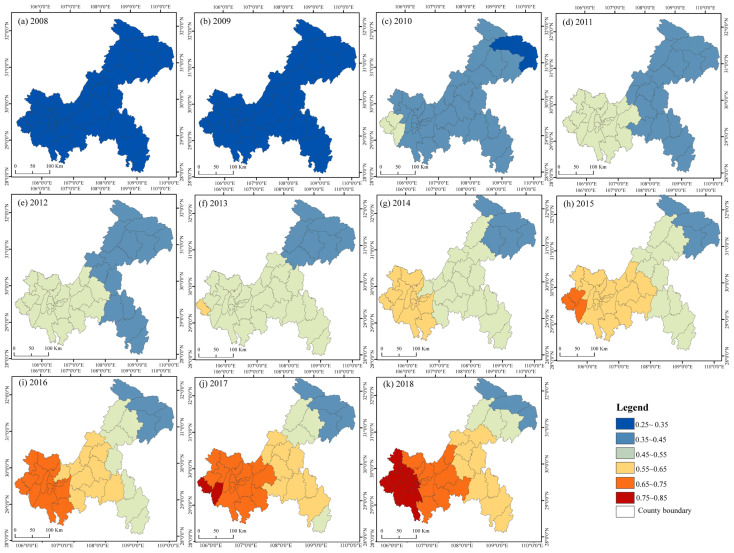
The spatiotemporal differences in the impacts of PU on LU. (**a**–**k**) The spatial distribution of the correlation coefficients of PU in GWR (2008–2018).

This paper uses GWR tools to reflect the LU affected by PU in time and space differentiation. We regard the rate of LU as the dependent variable and the rate of PU as the independent variable. After GWR, each district and county had a specific regression coefficient and a standardized residual value, through which the strengths and weaknesses of the model could be tested. We found that the range of residual errors of the remaining districts and counties were located in [−2.58 and 2.58], except Yuzhong, where the annual residual errors were all greater than 2.58, indicating that almost all the districts and counties passed the residual test. The overall fitting effect of the model was good. The GWR model calculates the unique regression coefficient under the impact of PU on LU in each district and county to intuitively present the spatiotemporal differences in the impacts of PU on LU across districts and counties (Figure 13).

From 2008 to 2018, the PU and LU maintained a positive correlation. From the spatial perspective, the impact of PU showed a decreasing trend from southwest to northeast. The influence of PU on LU was different in two town groups, decreasing from southwest to northeast in the UTA and decreasing from northwest to southeast in the UWA. The increasing speed of the impact of PU on LU showed different features among different regions. The correlation coefficient between PU and LU was less than 0.5 before 2011, but Yongchuan became the first district with a coefficient greater than 0.5 in 2011. In 2012, the PU coefficients were all greater than 0.5 in the western regions of the CP, including Dazu, Tongnan, Tongliang and Jiulongpo in the CUA. The areas with PU coefficients greater than 0.5 covered all of the CP except Changshou and Fuling in 2013, but the PU coefficients in the UTA and UWA were lower than 0.5. In 2014, the impact of PU on LU gradually expanded to the west. The urbanization coefficients of the whole region in the CP were all higher than 0.5. The urbanization coefficients of the districts and counties adjacent to the CP were also higher than 0.5, as in Dianjiang, Fengdu and Wulong. In 2015, the trend of the strong effect of PU on LU extended into the CP and the UWA, and the coefficients of Dianjiang, Fengdu, Liangping and Zhongxian in the UTA were also greater than 0.5. In 2016–2017, the coefficient in the CUA was greater than 0.6, which indicated that the impact of PU on LU was further strengthened. In 2018, only the PU coefficients in Chengkou, Wushan, Wuxi, Fengjie and Yunyang did not reach 0.5, but an increasing trend was still evident year by year compared with 2008.

## 4. Discussion

### 4.1. The Urban Land Expansion in Chongqing

From 2008 to 2018, the built-up areas of Chongqing continued to expand, with the highest annual growth rate of the built-up area in the NUA and the lowest annual growth rate in the UTA. The average annual growth rates of the NUA and CUA were higher than those of the two town groups. The urban expansion rates of each district and county were different, and the total amount of urban expansion was different to a certain extent: Dazu had the highest annual average growth rate of 15%, while Yuzhong had the lowest annual average growth rate of 1%. In 2008, China changed its land regulation policies from control to financial protection and implemented a stimulus plan of CNY 4 trillion [47]. It also adopted measures such as expanding domestic demand and joining investment to deal with the 2008 global financial crisis, which promoted urban land expansion from 2008 to 2011 to a certain extent. The expansion rate of built-up areas in Chongqing was generally higher than the growth rate in 2008–2018. After 2012, the economic growth changed to medium-high growth with the decline in China’s GDP. At this time, the expansion rate of the built-up area in the CUA gradually slowed, while the NUA, UTA, and UWA still maintained a relatively high growth trend. With China’s new-type urbanization in 2014, Dazhu, Yongchuan, Bishan, Tongnan, etc., became pilot areas. The growth rate of most built-up areas in the CP decreased significantly. The 2014–2020 New Urbanization Plan issued a policy description on the demarcation of the urban growth boundary and effectively solved the dilemma of the local government’s financial dependence on land [48]. According to the 13th Five-Year Plan for Land and Resources proposed by the Ministry of Land and Resources, the total amount of construction land should be controlled effectively from 2015 to 2020. The growth rate of built-up areas in Chongqing generally presented a downward trend since the newly increased construction land of China in 2015–2020 should be 4.46 × 10^5^ hm^2^ less than that in 2010–2015.The growth rate of built-up areas in Chongqing from 2014 to 2018 was generally lower than that in the previous two periods. In general, although the rate of built-up areas in Chongqing was still higher than the growth rate of the urban population, the expansion rate of built-up areas also slowed under the restrictive land policy. This reflects the importance of land and economic policy for land use change [49,50].

### 4.2. The Urban Population Growth in Chongqing

From 2008 to 2018, the urban population of most districts and counties increased, with the highest annual growth rate in the UWA and the lowest annual growth rate in the NUA. During this period, the urban population growth rate of the UTA and UWA was higher than that of the CP, completely different from the features of the expansion rate of built-up areas. Among the districts and counties, only Yuzhong experienced a decrease in the urban population. Bishan had the highest annual growth rate of 9%, and Jiangjin had the lowest annual growth rate of 1%, which reflects the spatial difference in urban population growth. The degree of urban agglomeration increased significantly from 2008 to 2015 and was controlled after 2015. In 2010, Chongqing took the lead in the reform of the household registration system (RHRS), resolving the long-standing social security problem that restricted nonurban people from settling in cities. We found that the urban population in each region of Chongqing increased rapidly from 2008 to 2011, at a rate higher than the average level in 2008–2018. Among them, the average growth rate of the urban population in Yubei reached an astonishing rate of 21% in 2008–2011. After 2008, under the development of western China and economic aggregate improvement, employment opportunities in the CUA of Chongqing gradually increased with the enhancing attractiveness of the city itself, and the urban population growth rate in the CUA further rose from 2011 to 2014. In 2013, the Chongqing municipal government proposed moderately reducing the population density of the core metropolitan functional area to alleviate the negative impact of urbanization. After 2014, the growth rate of the urban population in the CUA decreased slightly, which confirmed that the spatial agglomeration of the urban population declined slightly after 2015. The scale of the urban population in Yuzhong showed a declining trend in 2008–2018, which was considered to be a feature of urban shrinking under the circumstance of high population density.

### 4.3. The Spatial Heterogeneity of LU and PU

From 2008 to 2018, urban population growth and urban land expansion in Chongqing showed a rapid development pattern, but the growth rate of PU was lower than that of LU, presenting an uncoordinated state. Under the different space/time conditions, the coupling degrees were different. At the beginning of the study period, the unbalanced development relationship between population decrease and land increase appeared in the CUA, which gradually changed into the uncoordinated development mode of population and land increase. In the NUA, except Tongnan, which showed a balanced development trend before 2011, other areas maintained an uncoordinated trend of the simultaneous increase in population and land. Before 2011, the urban development in some areas of the UTA and UWA showed a coordinated trend of simultaneous increase in urban population and urban land. From 2011 to 2018, the coordinated development mode of simultaneous increase in urban population and land appeared only briefly in some districts and counties. The general development mode remained uncoordinated because LU increased faster than PU. The PU in Chongqing lagged behind LU, which was also the cause of insufficient population concentration in each district and county with low land use efficiency, extensive urban land use, and low land intensity. This paper posits that the urban–rural dual structure prevented the agricultural population from enjoying the same social welfare and security as the nonagricultural population when entering the urban built-up area [51], which hindered the speed of PU development. The government’s dependence on land finance led to the continuous expansion of urban built-up areas in Chongqing, resulting in the trend that the speed of LU was much higher than the speed of PU [52,53]. However, after the pilot reform of the urban–rural dual structure in 2010, Chongqing promoted PU. Moreover, the CUA became the first batch of new urbanization pilot cities, and the speed of urban land expansion was controlled to some extent, but the overall rate of LU was still much higher than the speed of PU.

### 4.4. The Spatial Heterogeneity of the Impact of PU on LU

The GWR model shows that PU significantly impacts LU in Chongqing, and the western area of Chongqing is more significantly affected by PU. However, at the local scale, the intensity and direction under the impact of PU on LU remained spatially heterogeneous. PU gradually became the critical factor to promote the process of LU, and this influence trend gradually spread to the east. As the central area of Chongqing’s urban development, the CUA retains a relatively strong foundation for PU. The PU mainly increased the demand for construction land in the city, further promoting the change in land use pattern, which became one of the main driving forces of LU development. In addition, the CUA took the lead in piloting new urbanization, and the adaptation degree of LU to PU led the upgrading of the area. However, the LU in the UTA and UWA was less affected by PU. As the urban development foundation in these areas was relatively weak, the PU in these areas remained at a lower level compared with the PU in the rest of the city, and the demands of construction land expansion had less impact on the local LU process. The process of LU was also affected by the natural geographical environment, social and economic demands, and other factors, so PU played a relatively minor role in the urban growth of these areas.

### 4.5. Gradual Coordinated Relation between LU and PU 

From 2008 to 2011, due to the small impact of PU on LU, the LU in the CUA maintained a higher growth rate than PU when the PU in the CUA declined or rose slowly. Because LU has no obvious response to PU, the PU growth rate was much faster than that of LU in some districts and counties in the UTA and UWA. The PU had little impact on LU, indicating that LU did not develop in accordance with the actual demand of PU, and there were situations of advanced development and unreasonable use of land resources, resulting in the waste of land resources. From 2014 to 2018, although the growth rate of LU was still higher than that of PU in most districts and counties, the gap between the two gradually narrowed. At this time, the coupling development coordination coefficient gradually became greater than 1, and the two progressively came to present coordinated development. At this stage, PU had an increasing impact on LU and became the main factor affecting LU, which indicates that urban land expansion takes the construction land demand under the urban population into consideration, gradually presenting a people-oriented development trend.

### 4.6. Deficiencies and Future Research 

The spatiotemporal heterogeneity of PU and LU is also affected by other spatial indicators, such as economic factors, external factors, and social factors [54]. This study aims to study urbanization features of Chongqing and provide a reference for rational land use, population policy setting, and new-type urbanization development. However, our study lacked consideration of other more in-depth spatial factors and elements of the polemics of the results obtained from research with similar results from other parts of the world. As a result, we need to improve the following aspects based on the existing research results: (1) In this paper, the long time series of urban built-up areas were extracted from NTL data that were preprocessed by radiation calibration. These data could support the study of urbanization at the county level and match the real built-up area more closely than statistical data do. With the increasing demand for urbanization research, it is worthy of further detailed study to use geographic information tools in order to extract the spatial form and internal differences in urban expansion in Chongqing. (2) Moreover, the coordination of the human–land relationship within the process of urbanization deserves further study in different areas of the world. In the urbanization scope of the Chengdu-Chongqing Economic Circle, it is necessary to explore the coordination and sustainable development of urbanization with the joint analyses of cities in Sichuan. Also, it would be worth proving and confronting with other examples in the world. (3) In future research, we could try to consider whether only the New Urbanization Plan affects the phenomenon under study, for instance the issue of the mechanism of land rent, suburbanization, the course of more important routes and transport nodes, growth poles, etc. Those indicators will be used to analyse the urbanization level by spatial econometric models, such as GWR, to better represent the spatial features of urban expansion and urban population growth.

## 5. Conclusions

As two major aspects of urbanization, it is particularly important to verify spatiotemporal heterogeneity in population growth and land expansion in the study of the sustainable development of urbanization. This study used a method based on NTL data and ISA data from 2008 to 2018 in order to extract continuous urban built-up areas, conducting qualitative and quantitative verification accordingly. We further studied the extracted built-up areas and urban population statistics to observe the spatial heterogeneity of urban land expansion and urban population growth from 2008 to 2018 by using the elasticity coefficient and GWR. Our results show that (1) the urban land of Chongqing expanded to 1.04 × 10^3^ km^2^ and the urban population increased by 6.8^5^ × 10^6^ people. Regardless of the built-up area, the total urban population of the CUA was much higher than that in the UTA and UWA. Nevertheless, the urban expansion rates in the CUA and NUA were faster than that in the two town groups, while the urban population growth rates of districts in the UWA were higher than that in the CUA, NUA, and UTA. (2) From 2008 to 2018, the average annual growth rate of urban land in Chongqing was 9.4%, which is higher than the urban population growth rate of 2.3%. The number of uncoordinated development types VI increased, which indicates that urban land expansion was extensive in Chongqing. (3) The impact of PU on LU reflected spatiotemporal heterogeneity, which had an increasing influence. The increasing influence promoted the relationship between LU and PU to become coordinated gradually. 

It is shown that Chongqing’s uncoordinated urbanization development modes also reflected the general situation of urbanization in the country [55]. After the RHRS and the adjustment of new pilot policies targeting the construction of new-type urbanization, LU and PU have gradually become coordinated with the urbanization development path. To solve the problem of uncoordinated PU and LU, Chinese urban areas should adhere to the principle of new-type urbanization construction and carry out scientific land planning strategies, strictly controlling land expansion to promote the reasonable development of population growth.

## Figures and Tables

**Table 1:** Basic information of the data used in this study.

Data Name	Data Description	Source
Defense Meteorological Satellite Program/Operational Linescan System (DMSP/OLS) nighttime light data	Annual nighttime light composite data with a spatial resolution of approximately 30 arc-seconds in 2008–2013.	The US Air Force Weather Agency and the Earth Observation Group of National Oceanic and Atmospheric Administration’s National Geophysical Data Center. (NOAA/NGDC) (https://www.ngdc.noaa.gov/eog/dmsp/DownloadV4composites.html, accessed on 1 March 2022)
National Polar—orbiting Partnership/Visible Infrared Imaging Radiometer Suite (NPP/VIIRS) nighttime light data	Annual nighttime light composite data with a spatial resolution of approximately 15 arc-seconds in 2013–2018.	NOAA/NGDC (https://www.ngdc.noaa.gov/eog/dmsp/downloadV4composites.html, accessed on 1 March 2022)
The impervious surface area (ISA) data	Average annual ISA data with a spatial resolution of 30 m in 2008–2018.	The China’s Tsinghua Database (http://data.ess.tsinghua.edu.cn, accessed on 5 March 2022)
Population density data	Accurate population data with a spatial resolution of 100 m in 2008–2018.	The WorldPop project at the University of Southampton in the UK by large-scale data processing in Microsoft Azure (https://www.worldpop.org/geodata/listing?id=69, accessed on 13 April 2022)
Statistical data	Annual statistical data at the prefecture level: the permanent urban population (10^4^) and urban area and built-up town area (10^4^ km^2^) in 2008–2018.	Chongqing Statistical Yearbook
Administrative boundaries	The administrative boundary vector data of districts and counties of Chongqing in 2008–2018	National Geomatics Centre of China (http://www.ngcc.cn/ngcc/, accessed on 1 March 2022).

**Table 2:** Temporal dynamics of the urban land increase rate in Chongqing (%).

Districts & Counties	2008–2011	2011–2014	2014–2018	2008–2018
BaNan	12.33	7.93	11.07	10.51
BeiBei	17.89	11.96	10.18	13.03
BiShan	12.66	10.79	8.48	10.43
ChengKou	—	3.25	9.35	4.72
DaDuKou	8.52	3.84	4.73	5.60
DaZu	13.72	22.92	10.31	15.12
DianJiang	10.13	11.40	7.38	9.41
FengDu	b3.76	4.93	3.08	3.84
FengJie	4.14	9.56	10.15	8.17
FuLing	11.64	15.56	8.20	11.44
HeChuan	13.73	13.44	8.47	11.54
JiangBei	14.91	7.69	5.88	9.13
JiangJin	19.57	11.10	10.70	13.48
JiuLongPo	10.89	6.26	6.80	7.86
KaiZhou	0.40	2.36	14.13	6.48
LiangPing	1.44	8.96	8.93	6.69
NanAn	9.55	7.27	6.54	7.66
NanChuan	10.66	17.09	7.25	11.23
PengShui	6.43	12.29	8.11	8.86
QiJiang	14.65	12.89	12.54	13.28
QianJiang	10.11	9.48	4.89	7.83
RongChang	8.89	8.23	9.75	9.03
ShaPingBa	14.45	6.45	5.88	8.62
ShiZhu	9.65	10.75	4.46	7.90
TongLiang	12.18	20.41	10.08	13.81
TongNan	10.95	12.96	10.03	11.19
WanZhou	0.98	1.71	6.06	3.23
WuShan	7.83	16.10	13.44	12.55
WuXi	5.34	22.83	18.57	15.88
WuLong	13.12	24.62	6.78	14.03
XiuShan	3.09	9.64	7.29	6.74
YongChuan	10.68	11.20	12.02	11.37
YouYang	7.84	9.43	5.37	7.33
YuBei	14.63	7.74	9.86	10.65
YuZhong	2.31	0.41	1.20	1.30
YunYang	2.66	6.71	6.58	5.44
ChangShou	18.23	14.55	6.13	12.29
ZhongXian	3.95	8.59	4.50	5.56

**Table 3:** Annual average growth rate of Chongqing’s urban population (%).

Districts & Counties	2008–2011	2011–2014	2014–2018	2008–2018
BaNan	4.37	4.29	3.79	4.11
BeiBei	1.67	3.39	3.66	2.98
BiShan	10.69	9.05	8.24	9.22
ChengKou	10.90	5.28	4.82	6.78
DaDuKou	4.40	4.67	2.90	3.88
DaZu	2.43	5.41	5.69	4.63
DianJiang	3.85	2.99	2.78	3.16
FengDu	8.24	2.44	1.74	3.90
FengJie	5.24	2.34	2.40	3.23
FuLing	3.45	2.85	3.33	3.22
HeChuan	3.81	1.06	1.00	1.86
JiangBei	1.35	4.24	2.26	2.58
JiangJin	0.70	1.84	1.79	1.48
JiuLongPo	0.63	5.29	3.95	3.36
KaiZhou	5.98	4.30	3.63	4.54
LiangPing	2.72	2.84	3.30	2.99
NanAn	2.14	6.01	5.41	4.61
NanChuan	2.12	3.30	3.18	2.90
PengShui	9.13	8.85	5.44	7.57
QiJiang	2.00	2.88	3.60	2.90
QianJiang	8.49	5.82	5.69	6.57
RongChang	5.60	3.93	4.58	4.69
ShaPingBa	2.63	6.03	4.12	4.25
ShiZhu	12.58	6.52	4.91	7.70
TongLiang	5.04	5.74	4.80	5.15
TongNan	7.91	5.54	4.95	6.01
WanZhou	4.09	2.93	2.13	2.96
WuShan	6.99	3.66	2.76	4.30
WuXi	7.81	3.68	3.44	4.82
WuLong	6.34	4.00	3.50	4.50
XiuShan	10.71	4.16	3.75	5.96
YongChuan	5.68	2.71	3.54	3.93
YouYang	8.82	8.34	5.94	7.52
YuBei	20.53	9.02	6.55	11.49
YuZhong	–3.97	–0.60	–0.90	–1.73
YunYang	2.63	6.52	4.86	4.69
ChangShou	3.65	2.22	1.54	2.38
ZhongXian	6.23	3.60	3.23	4.24

## Data Availability

No new data were created or analyzed in this study. Data sharing is not applicable to this article.

## Abbreviations

OATG	One Urban Area and Two Town Groups
CUA	The central urban area of Chongqing
NUA	The new downtown urban area
CP	The city proper of Chongqing
UTA	The urban clusters of Three Gorges Reservoir Area
UWA	The urban clusters of Wuling mountainous area
RHRS	The Reform of the Household Registration System
PU	Population urbanization
LU	Land urbanization
NTL	Nighttime light
GUR	The global urbanization region
